# Total mesorectal excision laparoscopic versus transanal approach for rectal cancer: A systematic review and meta-analysis

**DOI:** 10.1016/j.amsu.2022.103260

**Published:** 2022-01-24

**Authors:** Salvatore Lo Bianco, Katia Lanzafame, Caterina Domenica Piazza, Vincenzo Gaetano Piazza, Daniele Provenzano, Diego Piazza

**Affiliations:** a Department of Oncological Surgery, ARNAS Garibaldi Catania, Via Palermo 636, 95100, Catania, Italy; b Resident in Oncology, University of Messina, Piazza Pugliatti 1, 98122, Messina, Italy; c Department of MCAU, ASP Catania, Via Santa Maria la Grande 5, 95100, Catania, Italy

**Keywords:** TME, TA-TME, LA-TME, Rectal cancer, Mesorectum

## Abstract

**Introduction:**

Total mesorectal excision (TME) performed for the first time by Held through an open approach, it has become the standard technique for the surgical treatment of rectal cancer. The aim the of this meta-analysis is to compare the outcomes provided by TaTME than LaTME.

**Material and methods:**

In this meta-analysis, we included all comparative studies, prospective and retrospective, which addressed in low and middle rectal cancer, a comparison between TaTME and LaTME. A search was performed through MEDLINE and Cochrane Database. 846 records were identified.

**Results:**

Eight relevant studies have been included in this meta-analysis. The studies were from France, Russia, USA, Netherlands, Taiwan, Egypt. The eight studies including 471 patients with middle or low rectal cancer.

**Conclusion:**

The meta-analysis confirmed safety of TaTME for low and middle rectal cancer. TaTME can lead to a high quality of rectal cancer resection specimen.

## Highlights

• The aim the of this meta-analysis is to compare the outcomes provided by TaTME than LaTME.
• We included all comparative studies, which addressed in low and middle rectal cancer.
• Eight relevant studies have been included.
• The meta-analysis confirmed safety of TaTME for rectal cancer.

## 1 Introduction

Rectal cancer is one of the most common types of carcinoma throughout the world [1]. Over the years many techniques and technologies have been discovered to improve the patient's quality of life and the oncological outcomes associated with this pathology. The twentieth century, precisely 1907, marks the year in which Miles performs the first rectal surgery with radical intent. Total mesorectal excision (TME) performed for the first time by Held through with open approach, it has become the standard technique for the surgical treatment of rectal cancer [2]. In recent times, TME has shifted from the open approach to a laparoscopic technique (LaTME) [3]. The utility of LaTME is limited in patients with low rectal cancer, who require surgeons with experience in ultra-low sphincter-saving laparoscopic surgery, which has a high risk of leaving a positive circumferential resection margin [4].

Other factors, such as a narrow, irradiated pelvis and obesity, also predict intra-operative difficulties [5]. Lacy et al. Have reported the first case of Transanal TME (TaTME) in 2010 with satisfactory perioperative, pathologic, and oncologic results [6]. The aim of this meta-analysis is to identify the better outcomes provided by TaTME in comparison with LaTME in the treatment of low or middle rectal cancer.

## 2 Material and methods

This review has been reported in line with PRISMA (Preferred Reporting Items for Systematic Reviews and Meta-Analyses) [7] and AMSTAR (Assessing the methodological quality of systematic reviews) Guidelines.

### 2.1 Search methods for identification of studies

In this meta-analysis, we included all comparative studies, prospective and retrospective, which addressed in low and middle rectal cancer, a comparison between TaTME and LaTME.

A search was performed through MEDLINE and Cochrane Database using a combination of key terms: “transanal total mesorectal excision versus laparoscopic total mesorectal excision”, “transanal total mesorectal excision”, “laparoscopic total mesorectal excision”, “LaTME” and “TaTME”. 846 records were identified.

### 2.2 Inclusion and exclusion criteria

Studies were considered eligible in our meta-analysis if they met the following criteria: middle or low rectal cancer; surgical treatment for rectal cancer (taTME/laTME); comparative studies of TaTME with LaTME; comparison between groups of intraoperative data, postoperative, and oncologic results; and a study design such as prospective cohort study, case matched control study, and retrospective study.

The exclusion criteria were: inappropriate study design (review articles, non-English language studies, case report, nonhuman record, conference abstracts, letters to editor, and ongoing randomized trial), no LaTME control group, noncomparative studies, and duplicate publication or provision of insufficient data.

### 2.3 Data collection and analysis

The author (SL) reviewed all the eligible studies, according to the inclusion and exclusion criteria.

**Fig. 1:**
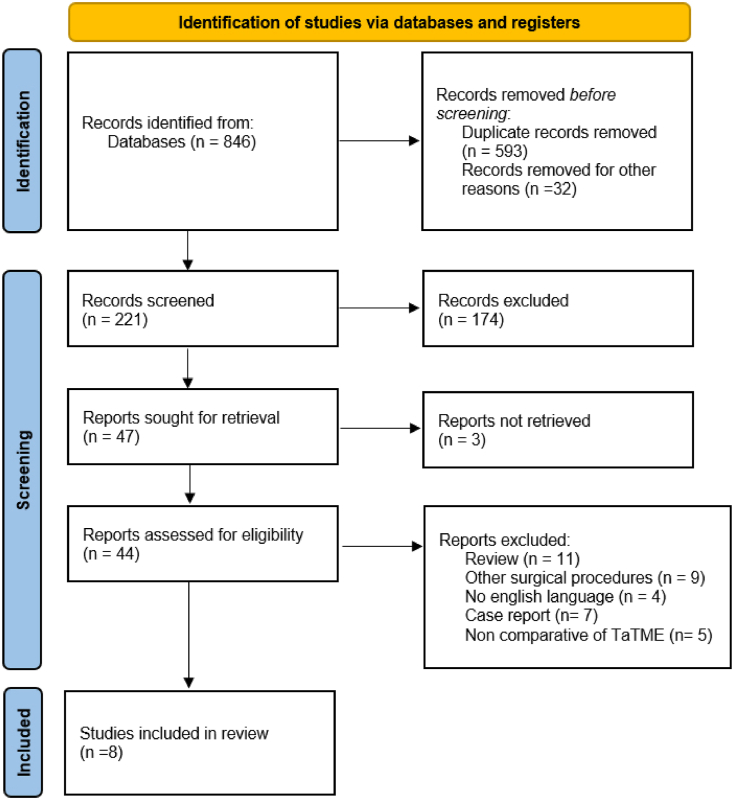
PRISMA flow chart.

**Fig. 2:**
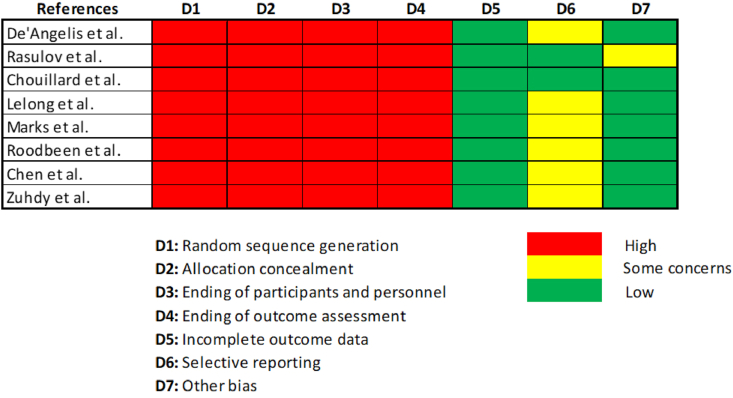
Newcastle–Ottawa Scale.

The search strategy was illustrated in the PRISMA flow chart (Fig. 1). The following information was collected: first author, year of publication, country, study type (RCT/cohort trial/matched case–control trial, etc.), number of patients enrolled, sex, age, tumor site (middle/low), surgical type of intervention, quality of mesorectum, positive circumferential resection margin (PCMR), operation time, hospital stay, anastomotic leakage, overall morbidity. The Newcastle–Ottawa Scale (NOS) criterion was used to evaluate the quality of the studies included (Fig. 2).

### 2.4 Statistical analyses

The statistical softwares Statistica v. 10.1020 and Comprehensive Meta Analysis v. 3 were used. Pooled odds ratios (OR) or weight mean differences (WMD), with their 95% confidence intervals (95%CI) were calculated for dichotomous or continuous variables, respectively. P value threshold for statistical significance was set at 0.05.

## 3 Results

### 3.1 Selected studies

The search strategy identified 846 studies (MEDLINE, Cochrane). After exclusion, 8 relevant studies have been included in this meta-analysis. The studies were from France, Russia, USA, Netherlands, Taiwan, Egypt [[8], [9], [10], [11], [12], [13], [14], [15]]. The eight studies including 471 patients with middle or low rectal cancer. 225 patients in the TaTME group and 246 in the LaTME group (Table 1). The overall mean of age is 65.04 ± 14.23 for LaTME group and 63.19 ± 14.73 for TaTME group (Table 2, Table 3). There was no significant difference between the two groups according to the age (95%CI, p = 0.38; participants = 471; studies = 8).

**Table 1:** Characteristics of included studies.

References	Year	Country	Type of study	Position of rectal cancer	Sample size (n)		Age mean (years)	
					LaTME	TaTME	LaTME	TaTME
De'Angelis et al.	2015	France	Case–control	Low	32	32	67.16	64.91
Rasulov et al.	2015	Russia	Cohort study	Low	23	22	60	56
Chouillard et al.	2016	France	Prospective cohort	Low	15	18	57.8	55.4
Lelong et al.	2016	France	Case–control	Low	34	38	56	54
Marks et al.	2016	USA	Case–control	Low	17	17	60	59
Roodbeen et al.	2018	Netherlands	Case–control	Low	41	41	66	62.5
Chen et al.	2019	Taiwan	Case–control	Low	64	39	64	62
Zuhdy et al.	2020	Egypt	Prospective cohort	Middle - low	20	18	53.40	53.89

**Table 2:**
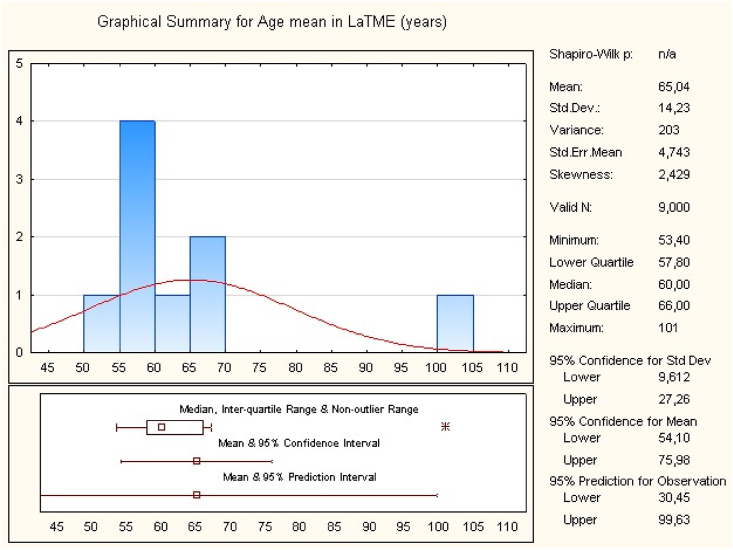
Overall mean of age for LaTME.

**Table 3:**
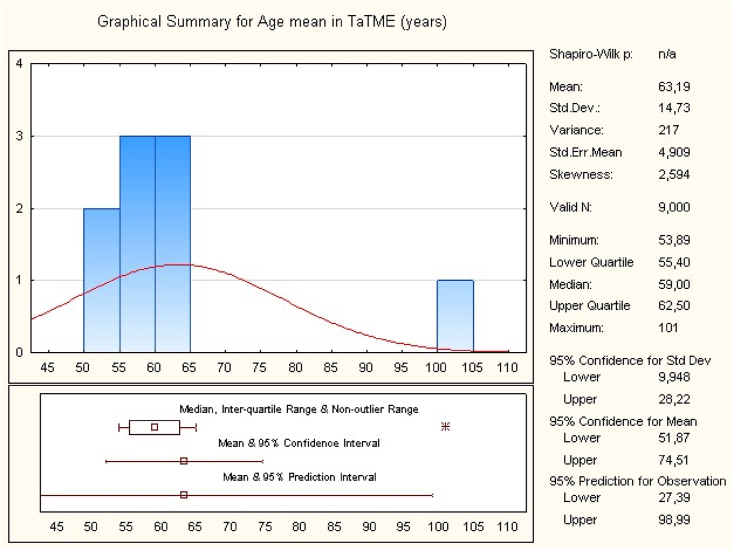
Overall mean of age for TaTME.

### 3.2 Outcomes

The main collected data from the eight studies are summarized in Table 4. Averages were collected for age, operative time and length of hospital stay. The number of events has been collected for the overall morbidity and positive circumferential resection margin (PCMR).

**Table 4:** Main collected data from the studies.

References	Operative time (min)				Overall morbidity (n)		Anastomotic leakage		Length of stay (days)				Positive circumferential resection margin	
	LaTME	SD	TaTME	SD	LaTME	TaTME	LaTME	TaTME	LaTME	SD	TaTME	SD	LaTME	TaTME
De'Angelis et al.	225	51.74	195	43.62	12	8	7	4	9.75	3.97	7.78	2.12	3	1
Rasulov et al.	305	59	320	68	4	6	1	0	8	2.43	8	2.49	0	1
Chouillard et al.	275	58	245	66	3	6	1	1	9.4	3.35	10.4	4.03	2	1
Lelong et al.	576	69	532	78	14	11	6	2	9	2.98	8	2.41	1	0
Marks et al.	380	62	421.7	73	5	4	0	0	5	1.96	5	1.92	0	0
Roodbeen et al.	300	58	318	67	14	19	4	5	11	4.22	8	2.99	5	1
Chen et al.	184	55	210	57	7	4	0	1	9.6	4.6	9.2	2.7	5	0
Zuhdy et al.	251.45	77.51	320.94	80.01	5	8	1	1	6	2.16	8	2.47	0	1

**Table 5:**
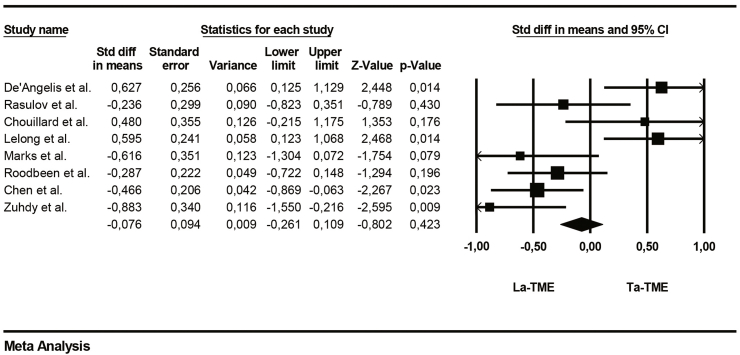
Forrest plot of operative time.

The operative time was shorter in the La-TME group than in the Ta-TME group, but the difference was not significant between the two groups (95%CI, p = 0.42; participants = 471; studies = 8; Table 5).

The length of stay was significantly shorter in the TaTME group than in the LaTME group (95%CI; p = 0.02, participants = 471; studies = 8; Table 6).

**Table 6:**
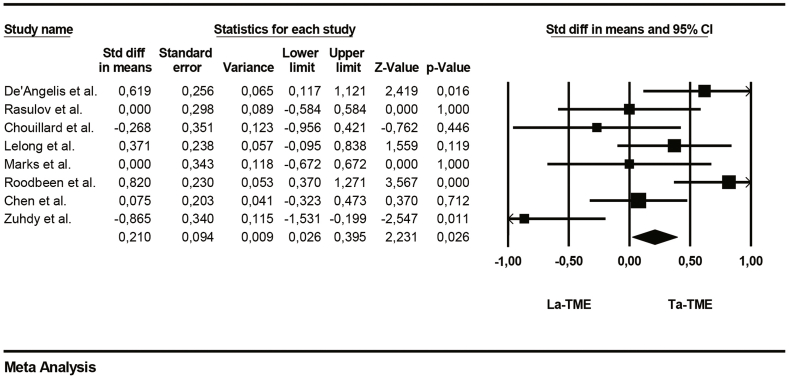
Forrest plot of Length of stay.

The incidence of overall morbidity is the same between the two groups and the difference is not significative. (95%CI; p = 0.73; participants = 471, studies = 8; Table 7).

**Table 7:**
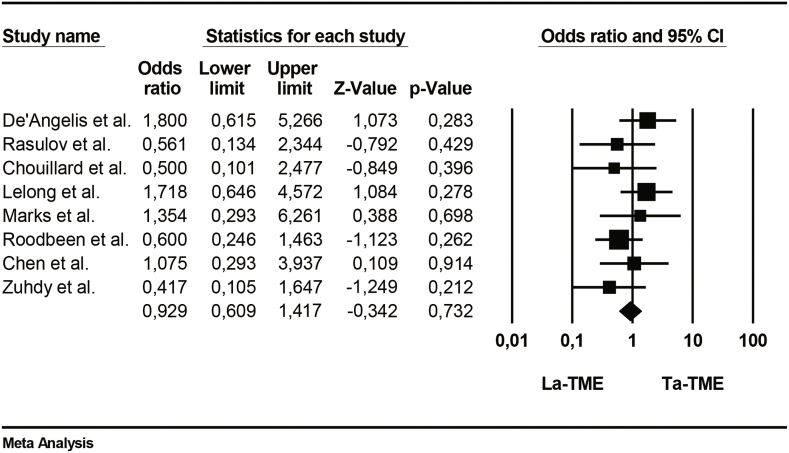
Forrest plot of Overall Morbidity.

When it was reported by the authors, major morbidity was mostly represented by anastomotic leakage. Anastomotic leakage was reported in 7 studies and occurred less frequently after TaTME than after LaTME. This difference between the two groups was significative (95%CI; p = 0.037; participants = 437;Table 8).

**Table 8:**
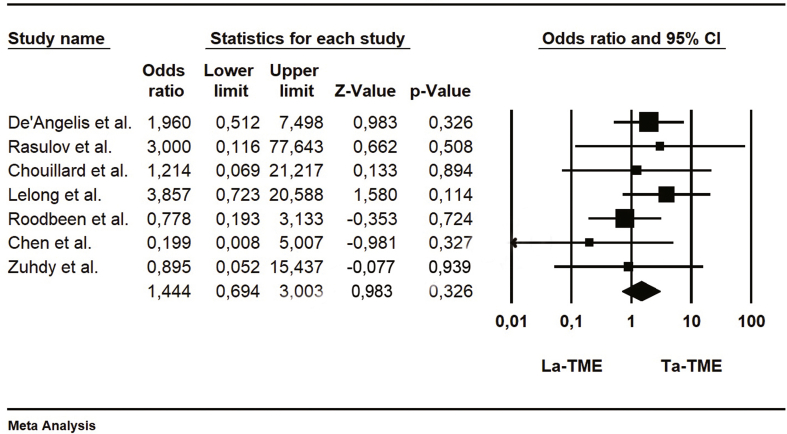
Forrest plot of Anastomotic leakage.

The positive involvement of circumferential resection margin (CRM) was reported in 7 studies and it was defined as the presence of tumor cells located ≤1 mm from the radial margin. TaTME was less frequently associated with positive CRM involvement than LaTME, and this difference was significative (95%CI, p = 0.049; participants = 437; Table 9).

**Table 9:**
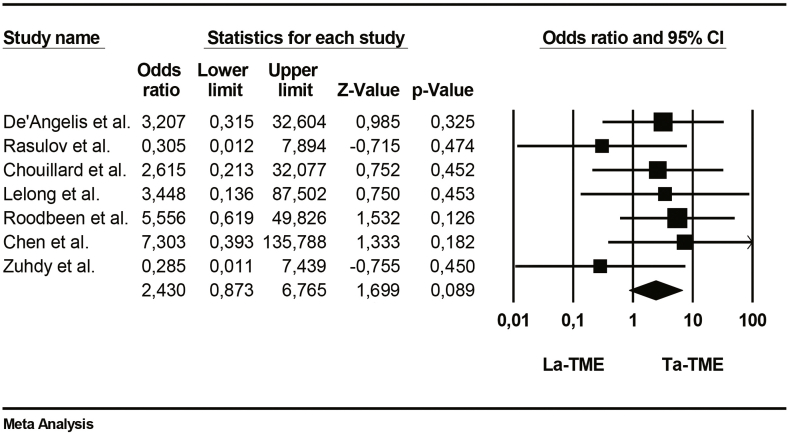
Forrest plot of Positive circumferential resection margin (PCRM).

All p-values of the examined parameters are shown in Table 10.

**Table 10:** p values.

Parameters	p-value
Age mean	0.29
Operative time	0.42
Overall morbidity	0.73
Anastomotic leakage	0.037
Length of stay	0.02
Positive circumferential resection margin	0.049

## 4 Discussion

LaTME procedures are generally thought to have better outcomes than open procedures. However, recent two studies both confirmed that laparoscopic resection failed to meet the criterion for noninferiority for pathologic outcomes when compared with open section for rectal cancer patients [16,17]. Proctectomy can be very difficult to work in the pelvis with rigid instruments from angles that require complicated maneuvers. AlaCaRT and ACOSOG Z6501 indicated that a different platform, such as robotics or TaTME, will improve efficacy of minimally invasive techniques.

TaTME is a new minimally invasive procedure with essential aim of improving oncological treatment quality and avoiding pelvic nerve injury in patients with mi- or low-rectal cancer. It defines more precisely the distal resection margin and allows the surgeon to perform the deep pelvic dissection without the need for difficult retraction [18]. Since its first description 8 years ago [19], TaTME is more and more adopted and performed. For several surgeons, it may facilitate the pelvic dissection, especially in male obese patient, bulky tumor, and in case of previous radiotherapy. Heald defines TaTME as the new solution to old problems [20].

Systematic review of literature showed that TaTME was significantly associated with a shorter length of stay, lower overall and major postoperative morbidities, anastomotic leakage, readmission and positive circumferential and distal resection margin involvement rates. Complete or nearly complete mesorectal fascia is a positive prognostic factor. An incomplete fascia is associated with unfavourable oncological outcomes [21]. Hence, for patients with mid- or low-rectal cancer, taTME may achieve a complete or nearly complete resection of the mesorectum relative easily, compared with laTME. In fact, a higher quality of mesorectal resection will convert into longer survival. In addition, TaTME had significantly shorter operation times and lower conversion rate. For these reasons, today, many authors have chosen TaTME not only in selected difficult cases but also as the standard approach for all the patients with low and middle rectal cancer.

Adopting a robotics system for the transanal approach confers three primary advantages. Firstly, doing so improves ambidexterity when performing lateral dissection. Secondly, surgical fields are much steadier compared to those offered under traditional laparoscopy. Thirdly, additional ports can be inserted via the GelPOINT Path platform to allow access for traction assistance and smoke evacuation. It is also noteworthy that utilizing the Gel-POINT apparatus at the stoma site not only avoids creation of an additional incisional wound, but also leaves the abdominal area open to access by robotics arms. The mean operative time is generally longer in robotic system, most likely attributable to time spent transanally docking the robotics arms and in part due to the surgeon changing between abdominal and transanal positions several times during the operative procedure. One possible solution is to create two-team approach for r-taTME ultimately decreased operative times [22]. In addition, robotics arms remain limited in depth penetration during transanale approach. However, new robotics systems based on single port access will open even more frontiers for this approach. In the available comparative studies, the conversion rate, intraoperative and postoperative complication rates, quality markers of rectal cancer surgery (achieve complete mesorectum, adequate number of lymph nodes harvested, and negative resection margins) appeared low and similar between robotic and laparoscopic approach [23,24].

The greatest limitation of robotic system studies is its lack of long-term oncologic outcome follow-up. The postoperative period currently remains too short to gather objective data [25].

## 5 Conclusion

The meta-analysis confirmed safety of TaTME for low and middle rectal cancer. TaTME can lead to a high quality of rectal cancer resection specimen, with shorter length of stay than LaTME.

Operating time is shorter for the laparoscopic procedure (LaTME). Anastomotic leakage was occurred less frequently after TaTME. Overall morbidities is comparable between the two procedures. Regarding Circumferential Resection Margin, TaTME demonstrated a lower percentage of positive margin for cancer, than standard LaTME.

## Annals of medicine and surgery

The following additional information is required for submission. Please note that failure to respond to these questions/statements will mean your submission will be returned. If you have nothing to declare in any of these categories, then this should be stated.

## Ethical approval

No ethical Approval is required for systematic review and meta-analysis.

## Sources of funding

No funding for our research.

## Author contribution

Salvatore Lo Bianco devised the research, researched the data, wrote the manuscript.

Katia Lanzafame wrote the manuscript.

Daniele Provenzano wrote the manuscript.

Caterina Domenica Piazza wrote the manuscript.

Vincenzo Gaetano Piazza wrote the manuscript.

Diego Piazza devised the research.

## Research registration Unique Identifying Number (UIN)

The manuscript is a meta-analysis and review of previous trials already registered on the public registry. The manuscript is not a "first in man" work and it is not prospective but retrospective. The only registry that accepts meta-analysis is "Prospero" but it only accepts meta-analysis in progress, not already completed.

## Guarantor

Salvatore Lo Bianco.

## Provenance and peer review

Not commissioned, externally peer-reviewed.

## Declaration of competing interest

Authors have no conflicts of interest to disclose.
